# OMIP‐090: A 20‐parameter flow cytometry panel for rapid analysis of cell diversity and homing capacity in human conventional and regulatory T cells

**DOI:** 10.1002/cyto.a.24720

**Published:** 2023-02-05

**Authors:** Wladislaw Stroukov, Daniela Mastronicola, Caraugh Jane Albany, Zeynep Catak, Giovanna Lombardi, Cristiano Scottà

**Affiliations:** ^1^ “Peter Gorer” Department of Immunobiology, School of Immunology & Microbiological Sciences King's College London London UK; ^2^ British Heart Foundation Centre, School of Cardiovascular Medicine and Sciences King's College London London UK

**Keywords:** cell subpopulations, cell trafficking, helper T cells, inflammation, regulatory T cells, tissue homing

## Abstract

The panel was developed and optimized for monitoring changes in homing capacity and functional diversity of human CD4^+^ conventional and regulatory T cell subsets. The analysis was based on expression of only surface markers in freshly isolated peripheral blood mononuclear cells (PBMCs) to reduce at minimum any alteration due to permeabilization or freezing/thawing procedures. We included markers to assess the distribution of naïve and memory populations based on the expression of CD45RA, CCR7, CD25, CD28 and CD95 in both conventional and regulatory T cells. The identification of major functional subsets was performed using CCR4, CCR6, CCR10, CXCR3 and CXCR5. Homing capacity of these subsets to skin, airway tract, gut and inflammatory lesions could finally be assessed with the markers CLA, CCR3, CCR5 and integrin β7. The panel was tested on freshly isolated PBMCs from healthy donors and patients with allergic rhinitis or autoimmune disorders.

## 1 BACKGROUND

CD4^+^ T cells play a central role in adaptive immunity supporting an appropriate immune response to external insults by secretion of cytokines and stimulation of target cells such as CD8^+^ T cells, B cells and innate immune cells. CD4^+^ T cells consist of conventional T helper cells (T_conv_ or T_H_), which mediate immune responses and immune‐suppressive regulatory T cells (Treg) that inhibit proinflammatory effector functions and thereby limit excessive immune responses. Both, activated T_reg_ and T_conv_ cells further differentiate into a wide range of subsets with distinct functions and phenotypes, but exhibit considerable phenotypical and transcriptional overlap [1, 2, 3]. During homeostasis of the immune system T cells migrate between tissue sites via peripheral blood in order to encounter their cognate antigens [4, 5, 6]. In contrast, pathogenesis of autoimmune diseases can cause imbalance of these T cell subsets resulting in dysregulation and excessive reactivity of lymphocytes against autoantigens. Aberrant homing capacity of CD4^+^ T cells can lead to accumulation of lymphocytes at auto‐immune tissue sites, thereby further contributing to the loss of self‐tolerance and orchestrating chronic or relapsing inflammatory responses [7, 8].

**FIGURE 1:**
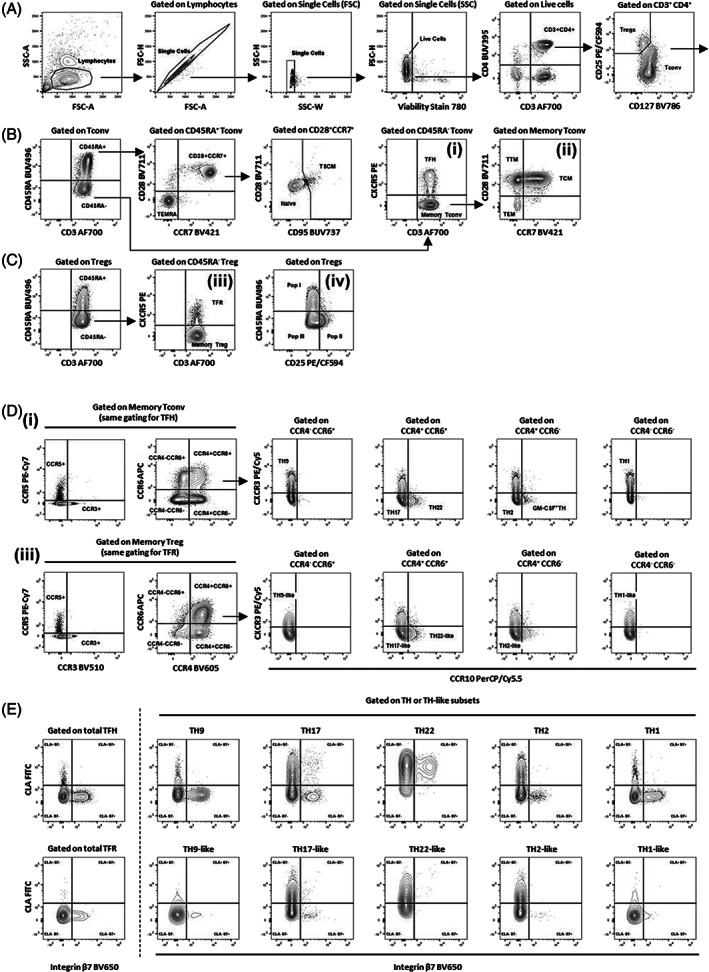
Gating strategy to identify viable T_reg_ and T_conv_ subpopulations from total PBMC. (A) Identification of T_reg_ and T_conv_ cells from viable CD3^+^CD4^+^ lymphocytes using surface markers CD25 and CD127. (B) T_conv_ were further separated in naïve, follicular helper T cells (T_FH_) and memory T_conv_ (i) through the expression of CD45RA, CCR7, CD28, CD95 and CXCR5. Memory T_conv_ were further separated (ii) in the other memory Tconv subsets (TSCM, TEMRA, TCM, TTM and TEM) using the expression of CCR7 and CD28. (C) Staining of CD45RA separated naïve T_reg_ (CD45RA^+^) from the other antigen experienced T_reg_ subsets. CD45RA^−^ T_reg_ were further separated through the expression of CXCR5 (iii) in memory (CD45RA^−^CXCR5^−^) and follicular (T_FR_, CD45RA^−^CXCR5^+^) cell subsets. Alternative gating (iv) based on the combination of CD45RA and CD25 on total T_reg_ could be used to identify the three functional subsets described by Miyara and collaborators. (D) Further gating of cells obtained in (i) (T_FH_ and memory T_conv_) or (ii) (T_FR_ and T_reg_) could be used to define the distribution of CCR3^+^, CCR5^+^ and T_H_ (or T_H‐like_) cells. (E) Analysis of skin and gut homing capacity of all the subsets described in panel B, C and D (T_FH_, T_FR_, T_H_ and T_H‐like_) using the expression of CLA and integrin β7.

Therefore, monitoring and a better understanding of phenotypic subset diversity and homing capacity of CD4^+^ T cell subsets during disease pathogenesis can contribute to informed therapeutical decisions and can improve clinical outcomes in patients. We developed a panel for monitoring common T_conv_ and T_reg_ cell subsets, including populations, which have been described in context of autoimmunity and their homing capacity to skin, gut, upper airways, and inflammatory lesions based on surface markers (Table 1 and Figure 1). To circumvent experimental complexity resulting from permeabilization and staining of intracellular transcription factors we focused our panel only on the staining of surface markers. This approach using combination of markers to identify CD4^+^ T cell subsets, allows to rapidly process large quantity of patient samples and makes the panel ideal for immune monitoring uses. Note, this panel is not limited to the investigation of autoimmune and inflammatory disorders, but also applicable for transplantation studies (where homing to tissue sites might affect outcome of transplantation). This panel is also compatible with live cell sorting for further investigation of functional properties of CD4^+^ T cell subsets.

**TABLE 1:** Summary table for application of OMIP‐090.

Purpose	Characterization of subset distribution and homing capacity in conventional and regulatory CD4^+^ T cells using only surface markers
Species	Human
Cell types	Fresh PBMC
Cross‐reference	OMIP‐017, OMIP‐018, OMIP‐030, OMIP‐042

Staining of viable cells for CD3, CD4, CD25 and CD127 allows to delineate between T_conv_ (CD25^−/+^) and T_reg_ cells (CD25^hi^ CD127^lo^), wherein the very high expression of CD25 combined to the low expression of CD127 describe a well‐defined population expressing high levels of the lineage‐defining T_reg_ marker FOXP3 [9]. Maturation of T_conv_ cells upon recognition of their cognate antigens results in differentiation from naïve to memory and effector subsets [10]. The use of markers like CD28, CD95 and CXCR5 along to the “classical” markers CD45RA and CD197 (CCR7) allows the identification of naïve and multiple memory/effector T_conv_ cell subsets. As a result, CD45RA^+^ cells can be separated into naïve cells (CD45RA^+^ CCR7^+^ CD28^+^ CD95^−^), T stem cell‐like memory cells which exhibit self‐renewal capacity (T_SCM_, CD45RA^+^ CCR7^+^ CD28^+^ CD95^hi^) [11, 12, 13] and terminally differentiated effector cells (T_EMRA_, CD45RA^+^ CCR7^−^ CD28^−^) [10].

Gating of CD45RA^−^ cells using the combination of CD3 and CXCR5 separates follicular helper T cells (T_FH_) from the other memory T cells. T_FH_ are a specialized cell subset that supports the antibody production against pathogens. The homing marker CXCR5 mediates the migration of these cells after recognition of antigens towards germinal centres, where they interact with B cells, undergo further differentiation, and provide B cell help for induction of antibody production [14]. The other memory T cells (CXCR5^−^) can be further separated using the expression of CCR7 and CD28 in the three main memory populations: central memory (T_CM_, CD45RA^−^ CCR7^+^ CD28^+^), transitional memory (T_TM_, CD45RA^−^ CCR7^−^ CD28^+^) and effector memory (T_EM_, CD45RA^−^ CCR7^−^ CD28^−^) T_conv_ cells.

Major T_H_ cell subsets can be identified from either T_FH_ (CXCR5^+^) or memory T cell (CXCR5^−^) populations according to lineage‐specific cytokine secretion and the chemokine receptors CCR4 (CD194), CCR6 (CD196), CCR10 and CXCR3 (CD183) [15, 16, 17]. T_H_1 cells (CCR4^−^ CCR6^−^ CCR10^−^ CXCR3^+^) are specified by the transcription factor T‐bet and produce the pro‐inflammatory cytokine IFN‐γ, activate macrophages and mediate immunity against intracellular pathogens. T_H_2 cells (CCR4^+^ CCR6^−^ CCR10^−^ CXCR3^−^) orchestrate defense against large extracellular pathogens and helminths by secretion of IL‐4, but are also involved in inflammatory disorders, such as asthma and allergies [18]. IL‐9 producing T_H_9 cells (CCR4^−^ CCR6^+^ CCR10^−^ CXCR3^+^) can be induced by the growth factor TGF‐β and IL‐4 from T_H_2 cells and are similarly involved in immunity against intestinal helminths and onset of allergies [19]. The transcription factor RORγT specifies the IL‐17 producing T_H_17 cell subset (CCR4^+^ CCR6^+^ CCR10^−^ CXCR3^−^), which mediate protection against fungi and extracellular bacteria [20, 21]. Further, balance of T_H_17 cells and T_reg_ cells plays a critical role in onset of autoimmune diseases [22, 23, 24]. Plasticity within the T_H_17 subset leads co‐expression of markers and functional properties that are shared between subsets. Indeed, in some conditions, RORγT^+^ T_H_17 cells can start expressing T‐bet, CXCR3 and secrete both IL‐17 and IFN‐γ. This subset (T_H_1‐like T_H_17 cells) might represent an intermediate and transitional state during trans‐differentiation between T_H_1 and T_H_17 cells [25]. T_H_22 (CCR4^+^ CCR6^+^ CCR10^+^ CXCR3^−^) are characterized by secretion of the eponymous cytokine IL‐22 and TNF‐α, and are involved in skin immunity and maintenance [26].

Similarly, T_reg_ cells can be separated into different stages of differentiation using CD45RA and CD25 markers. The level of expression of these two molecules can be used to identify the three functional subsets described by Miyara and collaborators: naïve (population I, CD45RA^+^ CD25^+^), effector (population II, CD45RA^−^ CD25^hi^) and cytokine‐producing (population III, CD45RA^−^ CD25^+^) T_reg_ cells [3, 27]. Besides, T_reg_ cells exhibit phenotypical and transcriptional overlap with the T_conv_ cells they target, therefore the same markers can be used to distinguish follicular regulatory (T_FR_) and T_H_‐like subsets within the T_reg_ compartment [1, 2, 28]. To explore subset diversity of T_reg_ cells, we set and adjusted the gates on clear populations of CD45RA^−^ memory T_conv_ cells across multiple donors and subsequently applied the same gates to either T_FR_ or memory T_reg_ cells.

Both chronic inflammatory diseases and autoimmune disorders can exhibit localized inflammation at various tissue sites. Inflammation is often mediated by the recruitment of T cells into these tissues. Migration is regulated by the expression of specific homing receptors that mediate rolling of T cells over activated endothelial cells and their trafficking into the tissue. We included the cutaneous lymphocyte antigen (CLA) as a crucial marker for initiation of skin homing of lymphocytes, which is associated with T cell mediated cutaneous inflammation [29, 30]. To identify lymphocytes homing to small intestine we used the expression of integrin β7 [31]. Staining of CCR3 for the recognition of T cells accumulating in the upper airway mucosa and involved in asthma and allergic inflammation [32, 33]. Finally, we also included the staining of CCR5 that is expressed on activated T cells involved in several pathological processes including autoimmunity, cancer, and HIV infection [34]. Its expression drives T cells to atherosclerotic plaques [35] and, in combination with CXCR3, to inflammatory lesions in autoimmune diseases such as rheumatoid arthritis (RA), inflammatory bowel disease (IBD) and multiple sclerosis (MS) [36, 37] (Table 2).

**TABLE 2:** Reagents used in OMIP‐090.

Reagent specificity	Clone	Fluorochrome	Purpose
CD3	UCHT1	AF 700	T cell Lineage
CD4	SK3	BUV 395	T helper Lineage
CD25	M‐A251	PE/CF594	T_reg_ Lineage
CD127	HIL‐7R‐M21	BV 786	T_reg_ Lineage
CD45RA	HI100	BUV 496	Naïve/memory
CD28	CD28.2	BV 711	Cell subsets
CD95	DX2	BUV 737	Cell subsets
CCR3	5E8	BV 510	Homing
CCR4 (CD194)	1G1	BV 605	Cell subsets
CCR5	J418F1	PE/Cy7	Activation/Homing
CCR6 (CD196)	G034E3	APC	Cell subsets
CCR7 (CD197)	G043H7	BV 421	Differentiation
CCR10	1B5	PerCP/Cy5.5	Cell subsets
CXCR3 (CD183)	1C6/CXCR3	PE/Cy5	Cell subsets
CXCR5 (CD185)	J252D4	PE	Homing
Integrin Beta 7	FIB504	BV 650	Homing
CLA	HECA‐452	FITC	Homing
Fixable Viability Stain 780	n/a	Live/Dead Near Infra‐Red (NIR)	Viability/Dump

## 2 SIMILARITY TO PUBLISHED OMIPS

This OMIP is similar to OMIP‐017, ‐018, ‐30 and ‐42 in their objective to identify multiple human T cells subsets in PBMCs using exclusively surface receptors [12, 15, 16, 17]. However, in OMIP‐17 and ‐18 there is no possibility to characterize any regulatory T cell subsets. The above similar OMIPs do not investigate cell trafficking to non‐lymphoid tissues (i.e., skin, gut, or airway mucosa).

## 3 ETHICAL USE OF HUMAN SAMPLES

All procedures performed on human participants were in accordance with the ethical standards of the Helsinki Declaration and ethically approved by HRA and Health and Care Research Wales (HCRW) with IRAS project ID 236524, REC reference 18/LO/1814. Informed consent was obtained from all individual participants involved in the study.

## CONFLICT OF INTEREST STATEMENT

The authors declare that the research was conducted in the absence of any commercial or financial relationships that could be construed as a potential conflict of interest.

### PEER REVIEW

The peer review history for this article is available at https://publons.com/publon/10.1002/cyto.a.24720.

## Supporting information


**MIFlowCyt:** MIFlowCyt Item Checklist

**Data S1:** Supporting Information
