# Supplementary material for: OMIP‐090: A 20‐parameter flow cytometry panel for rapid analysis of cell diversity and homing capacity in human conventional and regulatory T cells
Source: Cytometry A. 2023 Feb 5;103(5):362–7. doi: 10.1002/cyto.a.24720 (PMC10952450; doi:10.1002/cyto.a.24720)
Supplement: Supplementary file 1 — MIFlowCyt: MIFlowCyt Item Checklist [file CYTO-103-362-s002.docx]

**Cytometry Part A**

**Author Checklist: MIFlowCyt-Compliant Items**

| **Requirement** | **Please Include Requested Information** |
| --- | --- |
| 1.1. Purpose | This panel was developed and optimized for monitoring changes in homing capacity and functional diversity of human CD4^+^ conventional and regulatory T cell subsets. The analysis was based on expression of only surface markers in freshly isolated peripheral blood mononuclear cells (PBMCs) to reduce any alteration due to permeabilization or freezing/thawing procedures and perform the staining in short time.  We included markers to assess the distribution of naïve and memory populations (CD45RA, CCR7, CD25, CD28 and CD95) of both conventional and regulatory T cells along with the identification of their major functional subsets (CCR4, CCR6, CCR10, CXCR3 and CXCR5). Homing capacity of these subsets to skin, airway tract, gut and inflammatory lesions could also be assessed with the markers CLA, CCR3, CCR5 and integrin β7. The panel was tested on freshly isolated PBMCs from healthy donors and patients with allergic rhinitis or autoimmune disorders. |
| 1.2. Keywords | regulatory T cells, helper T cells, cell subpopulations, tissue homing, cell trafficking, inflammation |
| 1.3. Experiment variables | Antibody concentration, human helper and regulatory T cell subset distribution and homing capacity |
| 1.4. Organization name and address | King’s College London. Immunoregulation laboratory, 5^th^ floor, Bermondsey Wing, Guy’s Hospital. SE1 9RT |
| 1.5. Primary contact name and email address | Dr Cristiano Scottà, Cristiano.scotta@kcl.ac.uk |
| 1.6. Date or time period of experiment | July 2020- October 2022 |
| 1.7. Conclusions | The procedure described a rapid method to analyse homing capacity and functional heterogeneity of human regulatory and helper T cell subsets on freshly isolated PBMC by flow cytometry. |
| 1.8. Quality control measures | **Instrument QC:**  - All data were acquired on the same flow cytometer using the same application settings.  - Flow cytometer passed QC tests using BD™ CS&T beads before the sample acquisition to provide a standardized method to perform quality control of the instrument’s optics, electronics, and fluidics, and for adjusting fluorescence compensation.  **Sample QC:**  - All biological samples were processed and stained in the same way.  - Single antibodies were titrated using increasing concentrations (see Online Figure 1). Titrations were performed on the same number (1 million) and type of cells (human PBMC from healthy individual) used for the fully stained samples  **Data QC:**  **-** Data were analysed by the same person with uniform application of gating strategy across all samples. |
| 2.1.1.1. (2.1.2.1., 2.1.3.1.) Sample description | Human peripheral blood mononuclear cells (PBMCs) |
| 2.1.1.2. Biological sample source description | Human peripheral blood mononuclear cells (PBMCs) |
| 2.1.1.3. Biological sample source organism description | Human peripheral blood mononuclear cells (PBMCs) |
| 2.1.2.2. Environmental sample location | N/A |
| 2.3. Sample treatment description | PBMCs were isolated from whole blood by Ficoll-Paque gradient centrifugation and stained within 4 hours. For a detailed protocol see Online Supporting Information |
| 2.4. Fluorescence reagent(s) description | see Online Table 2 |
| 3.1. Instrument manufacturer | Becton Dickinson |
| 3.2. Instrument model | LSR Fortessa |
| 3.3. Instrument configuration and settings | see Online Table 1 (Instrument configuration) |
| 4.1. List-mode data files | Exemplary data files from 3 individuals (see online description) were submitted to [http://flowrepository.org](http://flowrepository.org/)  http://flowrepository.org/id/ FR-FCM-Z4C6  URL: <https://flowrepository.org/id/RvFrht4UEL8oNrHFtYFy8Ru0WJRhnnoHuSIMrcGsjmMW9hXn2SCJHYjekghh1TT1> |
| 4.2. Compensation description | Compensation was performed using BD DIVA software with single stained beads (all fluorochromes) or cells (viable dye and unstained control) prior to acquisition and adjusted using Flowjo software. |
| 4.3. Data transformation details | Bi-exponential transformation using Flowjo software. |
| 4.4.1. Gate description | see Online Table 3a and 3b (T cell populations identified by the panel) and Manuscript Figure 1 (Gating strategy and example staining) |
| 4.4.2. Gate statistics | Calculated by Flowjo software. |
| 4.4.3. Gate boundaries | Gate boundaries were set: 1) by hand, where there was a clear divide between positive and negative staining. Or 2) by using FMO controls for those cell population which did not show a clear separation between positive and negative cells. |

**Notes**

Feel free to use more space than allocated.

You can embed graphics/figures in this document, if needed.

Please make sure to save the document in Microsoft Word version 2003 or older, before uploading to ScholarOne Manuscripts. When uploading this checklist to ScholarOne Manuscripts, please choose the “Supplementary Material for Review” category.

Please note that if your paper is accepted, the checklist will be published as an Online Supporting Information.

For any questions, please contact the Cytometry Part A editorial office at [Cytometrya@wiley.com](mailto:Cytometrya@wiley.com).
