# Supplementary material for: OMIP‐090: A 20‐parameter flow cytometry panel for rapid analysis of cell diversity and homing capacity in human conventional and regulatory T cells
Source: Cytometry A. 2023 Feb 5;103(5):362–7. doi: 10.1002/cyto.a.24720 (PMC10952450; doi:10.1002/cyto.a.24720)
Supplement: Supplementary file 2 — Data S1: Supporting Information [file CYTO-103-362-s001.docx]

# Online Material for “A 20-parameter flow cytometry panel for rapid analysis of cell diversity and homing capacity in human conventional and regulatory T cells” manuscript by *Stroukov et al*.

# **Panel development**

## General considerations

The aim of this panel was to have a practical tool to perform an analysis on freshly isolated peripheral blood cells of both T_conv_ and T_reg_ functional subpopulations and their homing capacity. The goal was to have a panel to rapidly analyse a large quantity of samples from patients in clinical trials avoiding freezing procedures that may affect the expression of functional antigens. In order to reduce the time of sample processing and make the panel (or part of it) compatible with live cell sorting, we circumvented the use of intracellular markers which require cell fixation, permeabilization and longer staining procedures. We used differential expression of surface markers such as CCR4, CCR6, CXCR3, CD25 and CD127 in place of the intracellular staining of master gene regulators like T-bet, GATA3, RORɣT or FOXP3 to identify T_H_1, T_H_2, T_H_9, T_H_17, T_H_22 and T_reg_ cell subsets.

Finally, to maximise the detection of fluctuating chemokine receptors, we performed the staining at the physiological temperature of 37°C. In general, this approach helped to preserve the original expression of unstable antigens in a no labour-intensive procedure.

## Marker priority

Because the aim of this panel was to quantify changes in conventional T cell subsets and the corresponding regulatory T cell subsets, we assigned different priorities to the markers. Markers to separate live conventional and regulatory T cells were assigned the highest priority (viability, CD3, CD4, CD25 and CD127) followed by the combination of markers to separate naïve and memory cells (CCR7, CD28, CD95 and CD45RA) and markers to identify functionally defined memory subpopulations (CCR4, CCR6, CCR10, CXCR3, CXCR5). Finally, the tissue homing capacity of each defined subset was assessed by the expression of CLA, CCR3, CCR5 and Integrin β7.

## Choice of fluorochrome-marker combination

The pairing of the antigens with fluorochromes was based on the following criteria: (i). Antigen density on cell surface; (ii). Resolution necessary to successfully separate the population of interest; (iii). Importance of cell population and (iv). Cell distribution. In general, we chose very bright fluorochromes for lowly expressed markers that were difficult to separate from a negative population. For this reason, we tried to use the brightest fluorochromes (i.e., BV421, BV650, APC and PE or PE tandem dyes excited by the yellow laser) for such difficult markers (e.g., CCR6, CCR7, CXCR3 or CXCR5). Antibodies against antigens with a very high surface density such as CD3, CD4 and CD45RA were conjugated to moderate or dim fluorochromes. This approach helped with the photon-counting errors (spill-over spreading error or SSE) that can spread the signal of one channel into the others and limit the cell resolution. We tried as far as possible to pair fluorochromes with large SSE to markers that were not co-expressed on the cell subset of interest.

Different fluorochrome alternatives were tested to find the best combination fluorochrome-marker and improve the separation of cell populations which are critical for the further definition of cell subsets.

We started this process using as a backbone the combination of antibodies: anti-CD3 AF700 (UCHT1), anti-CD4 BUV395 (SK3), anti-CD28 BV711 (28.2), anti-CD45RA BUV496 (HI100) and Fixable Viability Stain 780. These antibodies were maintained associated to those fluorochromes in the whole multiple iterative progression required to achieve the best resolution for all the markers.

We proceeded using the following iteration process:

1. Selection of the best fluorochrome combination to identify the target cell population
2. Comparison of alternative clones to obtain the best resolution
3. Gating of the cell subset using FMO

Initially, we optimised the CCR4 and CCR6 staining on CD3^+^CD4^+^CD45RA^-^ cells. A good separation with these two markers is critical for the definition of both T helper and T helper-like subsets. We set the first staining with anti-CCR4 and anti-CCR6 antibodies that were already established in our lab. We tested anti-CCR4 (clone 1G1) conjugated to either BV605 or PE/Cy7 in combination with anti-CCR6 (clone G034E3) conjugated to FITC (see Online Figure 1). We realised that to achieve a good separation between CCR4^+^ and CCR6^+^ cells we needed a bright fluorochrome for CCR6. So, we tested anti-CCR6 antibody (clone G034E3) conjugated to BV786 and APC. Results in Online Figure 1 show that the best separation was achievable with anti-CCR6 conjugated to APC in combination with anti-CCR4 BV605. In an attempt to further improve CCR6^+^ cell separation, we also compared anti-CCR6 APC clone G034E3 with clone QA17A37 on three blood samples from different healthy donors. As shown in Online Figure 2A, clone G034E3 was slightly better than clone QA17A37 (Mean Fluorescence Intensity (MFI) 2449 vs 2120 and Stain Index (SI) 26,036 vs 22,527 respectively) and it was included in the panel.

Another combination of markers that required some optimisation was CCR7 and CD28. These markers expressed on CD3^+^CD4^+^CD45RA^+^ or CD45RA^-^ cells are crucial to define subsets of conventional T cells such as T_EMRA_, T_CM_, T_EM_, etc. We tested anti-CCR7 (clone G043H7) conjugated to PerCP/Cy5.5, PE/dazzle 594 and BV421 in combination with CD28 BV711 on both CD45RA^+^ and CD45RA^-^ T cells. Results in panel B of Online Figure 1 showed that the best cell separation was reached with CCR7 BV421. A similar outcome was obtained using an alternative and more conventional strategy to identify the same cell subsets by gating CD3^+^CD4^+^ cells using CD45RA and CCR7 (Online Figure 1, panel C).

The double staining with CD28 and CD95 on CD45RA^+^CD28^+^CCR7^+^ T cells identifies naïve and T_SCM_ cells. To optimise the separation between these subsets, we focussed on CD95 (clone DX2) staining and tested antibodies conjugated to both PE/Cy7 and BUV737. Results showed similar staining (Online Figure 1, panel D) and due to the limited number of commercially available markers conjugated to BUV737, we chose to use anti-CD95 associated to this fluorochrome.

Our previous experience showed that the staining of CXCR3^+^ cells also required a bright fluorochrome. We decided to use anti-CXCR3 conjugated to PE/Cy5 (excited by a yellow laser) produces a very good cell separation. To optimise this staining, we tested two different anti-CXCR3 PE/Cy5 clones: 1C6/CXCR3 (BD Biosciences) and G025H7 (Biolegend). Although, the staining on three samples from different healthy donors allowed to clearly define CXCR3^+^ cells with both clones, clone G025H7 produced the best cell separation as previously shown by Liechti T, Roederer M. in OMIP-051 (Cytometry A. 2019 Feb;95(2):150-155. doi: 10.1002/cyto.a.23689). Clone G025H7 was chosen for the staining of CXCR3 and included in the panel (Online Figure 2, panel B).

The staining of eotaxin receptor CCR3 on T cells does not always show a very defined population. So, to compare different antibodies and setup the staining we decided to use total lymphocytes. Due to the limited availability of clones conjugated to BV510 (antibodies exclusively commercialised by BD Biosciences and Biolegend), we tested only the clone 5E8. Cells from three different healthy donors were used to compare anti-CCR3 BV510 purchased from BD Biosciences and Biolegend. As expected, the results showed a very similar staining (Online Figure 2, panel C). We chose to include the Biolegend antibody in this panel.

The staining of CXCR5 was used to identify conventional and regulatory T cells trafficking to B cell zones in the lymph nodes. In our laboratory, we successfully used clone J252D4 from Biolegend, but found that the choice of the fluorochrome associated to this antibody can dramatically improve the quality of the staining. For this reason, we decided to use this antibody associated to a bright fluorochrome. In panel D of Online Figure 2, we show the comparison of the staining with clone J252D4 conjugated to PE and APC on CD3^+^CD4^+^CD45RA^-^ cells from three different healthy donors. Anti-CXCR5 PE allowed a better cell separation than the antibody conjugated to APC, and we selected the antibody conjugated to this fluorochrome to be included in the panel.

One of the main goals of this panel was the parallel analysis of T_reg_ and T_conv_ cells. A good staining of both CD25 and CD127 is necessary to separate these cells and allow the definition of their subpopulations. Based on previous experience, we used anti-CD25 conjugated to the very bright fluorochrome PE/CF594 that works very well in this setting. Instead, in an attempt to optimise the separation between T_conv_ and CD127^-/low^ T_reg_ cells, we compared two different anti-CD127 BV786 (clone HIL-7R-M21 from BD Biosciences and clone A019D5 from Biolegend). Panel E of Online Figure 2 shows the staining of CD3^+^CD4^+^ T cells from three different samples with these antibodies. The comparison revealed clone HIL-7R-M21 to be slightly better than clone A019D5 (MFI 425 vs 267 and SI 9,000 vs 5,047 respectively) that for this reason was included in the panel. To correctly separate T_conv_ from T_reg_ cells and define T_reg_ subpopulations, we used the following gating strategy based on the different expression of CD25 and CD127 on these two populations (see panel A of Online Figure 3). Fluorescence minus one (FMO) control for CD25 was used to discriminate CD25^-^ T_conv_ from CD25^+^ T_conv_ and T_reg_ cells. The separation of CD25^+^CD127^-/low^ T_reg_ from T_conv_ cells in the fully stained sample was straightforward with the T_reg_ cells form a clearly separated population from the other cells. Because the further delineation of T_reg_ subpopulations (especially Pop II from Pop III) can sometimes result problematic, we adopted the following approach: We split CD25^+^CD127^-/low^ cell subset in two parts (named ‘Treg’ and ‘CD25high Treg’ respectively in panel A of Online Figure 3) according to the top edge of CD25^+^ T_conv_ subset. This method allowed to find the position of the three subpopulations in the next CD45RA/CD25 dot plot. The position of Pop I (CD45RA^+^CD25^+^) and Pop III (CD45RA^-^CD25^+^) was easily defined using the ‘Treg’ gate and the position of Pop II (CD45RA^-^CD25^high^) was given by ‘CD25high Treg’ gate (see overlaying contour plot and corresponding gating in Online Figure 3, panel A).

To establish the correct gating strategy for the other cell subsets we performed some FMO controls including the isotype control for the corresponding ‘*minus* one’ antibody. This approach was particularly helpful to identify the spread of the negative population due to no specific antibody binding and define the boundaries between positive and negative populations. This strategy was used in the staining with CD28-CD95 (Online Figure 3, panel B), CCR5-CCR3 (Online Figure 3, panel C), CXCR3-CCR10 (Online Figure 3, panel D) and CLA-Integrin beta 7 (Online Figure 3, panel E) where the expression level of markers like CD95, CCR3, CCR10 and CLA can sometimes be substantially low.

## **Selection of the best fluorochrome combination - Online Figure 1**


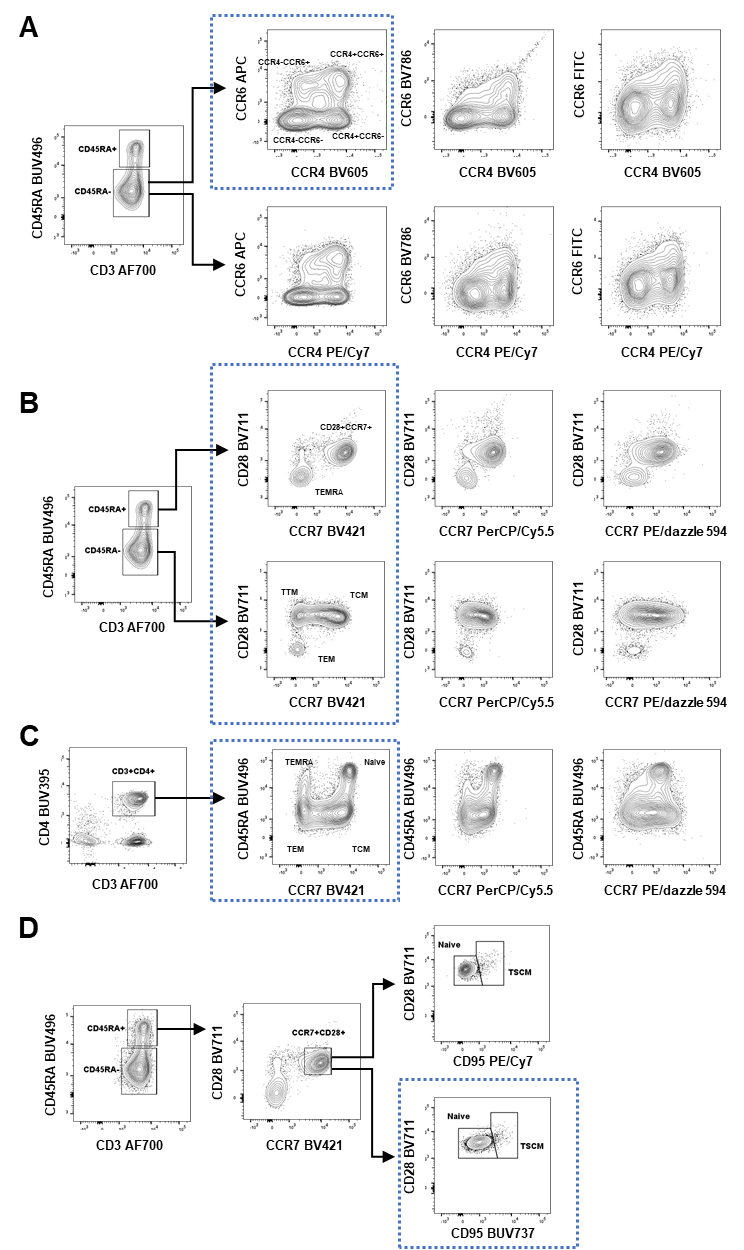


**Online Figure 1**. **Selection of the best fluorochrome combination to identify the target cell population.** (A). Optimisation of CCR4 and CCR6 staining combination on CD3^+^CD4^+^CD45RA^-^ cells comparing anti-CCR6 conjugated to APC, BV786 and FITC with anti-CCR4 conjugated to BV605 and PE/Cy7. (B). Optimisation of CCR7 and CD28 staining on both CD3^+^CD4^+^CD45RA^-^ and CD3^+^CD4^+^CD45RA^+^ cells. Anti-CD28 BV711 was tested in combination with anti-CCR7 conjugated to BV421, PerCP/Cy5.5 and PE/dazzle 594. (C). Anti-CCR7 conjugated to BV421, PerCP/Cy5.5 and PE/dazzle 594 was also tested in combination with anti-CD45RA BUV496 on CD3^+^CD4^+^ T cells as alternative approach to separate naïve and memory T_conv_ subsets. (D). Comparison of anti-CD95 conjugated to either BUV737 or PE/Cy7 to optimise the separation of naïve and T_SCM_ cells on CD3^+^CD4^+^CD45RA^-^CCR7^+^CD28^+^ cells. Blue round dotted squares identify the fluorochrome combination selected in the panel.

## **Antibody clone and fluorochrome comparison - Online Figure 2**


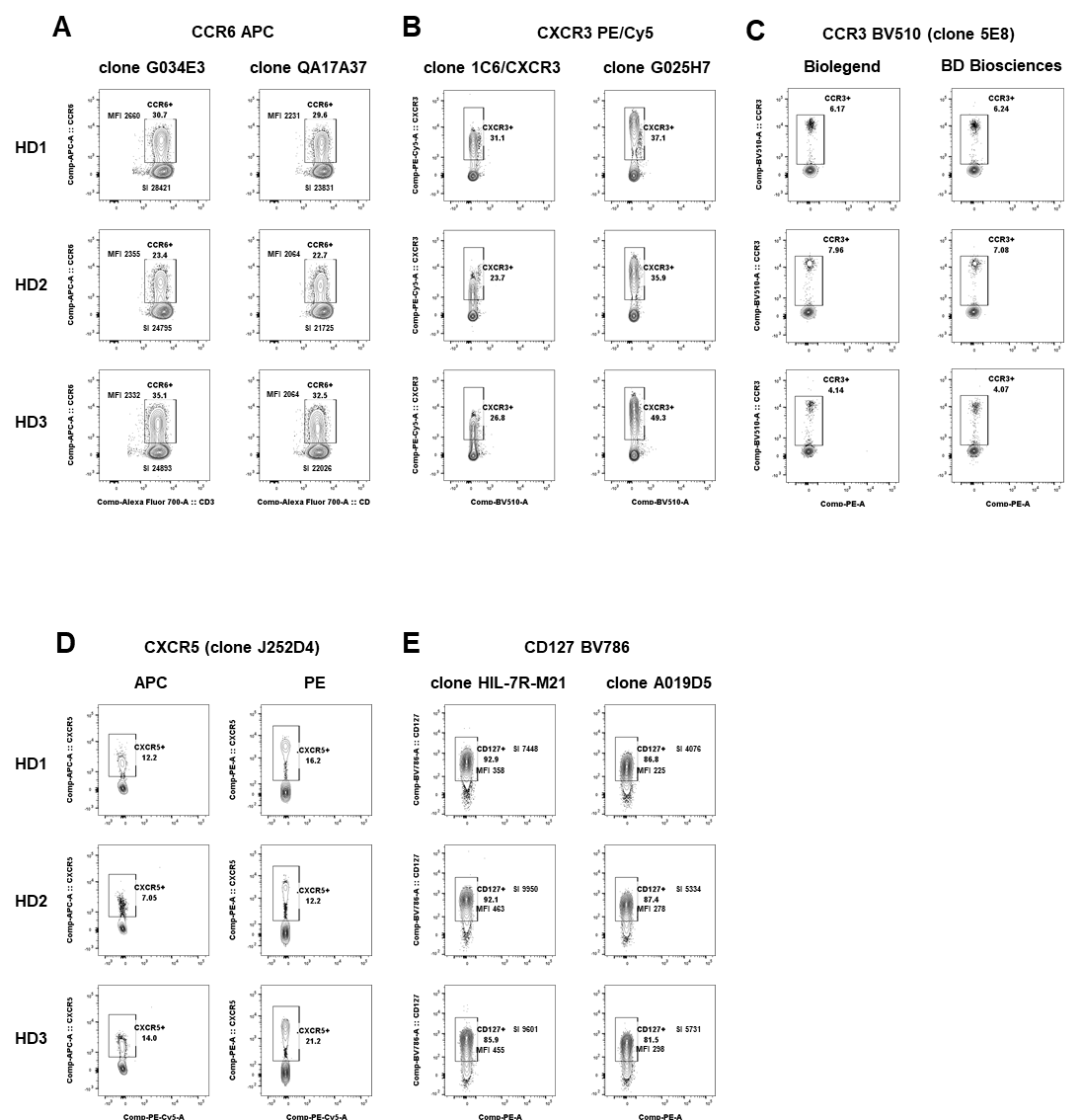


**Online Figure 2.** **Comparison of alternative clones and fluorochromes to obtain the best cell subset resolution.** (A). Comparison of G034E3 and QA17A37 clones conjugated to APC and recognising CCR6 molecule on memory T_conv_ from three different healthy controls. Numbers on the dot plot report percent of positive cells, MFI of positive population and stain index. (B). Comparison of 1C6/CXCR3 and G025H7 clones conjugated to PE/Cy5 and recognising CXCR3 molecule on memory T_conv_ from three different healthy controls. (C). Comparison of the same anti-CCR3 clone 5E8 conjugated to BV510 from Biolegend and BD Biosciences. Staining performed on memory T_conv_ from three different healthy controls. (D). Comparison of the same anti-CXCR5 clone J252D4 from Biolegend conjugated to either PE or APC. Staining performed on memory T_conv_ from three different healthy controls. (E). Comparison of HIL-7R-M21 and A019D5 clones conjugated to BV786 and recognising CD127 molecule on CD3^+^CD4^+^ T cells from three different healthy controls. HD: healthy donor, MFI: mean fluorescence intensity, SI: stain index.

## **Gating with FMO controls - Online Figure 3**


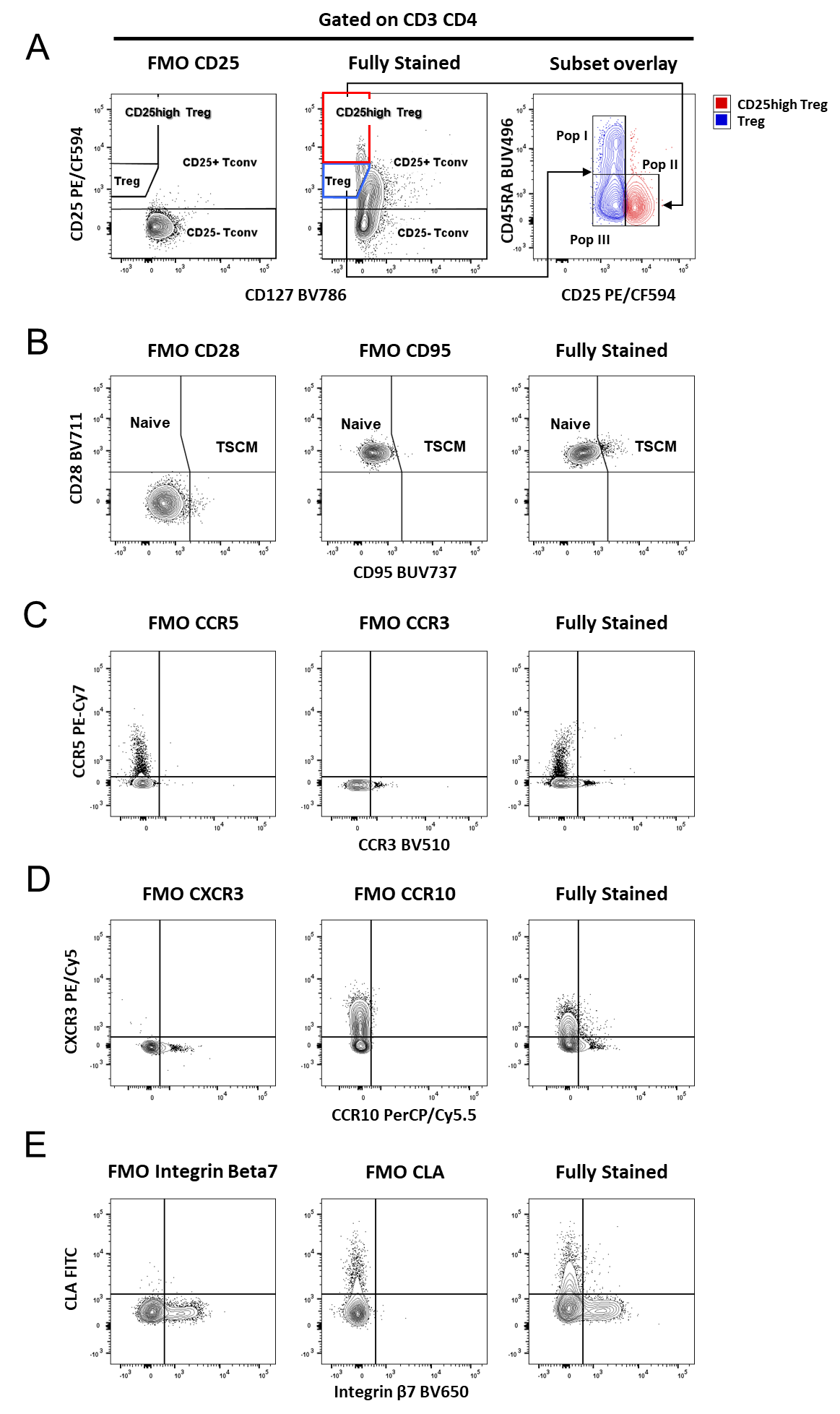


**Online Figure 3. Gating of the cell subset using FMO/isotype controls**. (A). CD25 FMO control to establish the gating strategy to separate CD25^-^, CD25^+^ T_conv_ and CD25^high^ T_reg_ cells. The definition of these subsets allowed the gating of Pop I, II and III of T_reg_ cells. (B). CD28 and CD95 controls to separate naïve from T_SCM_ cells. (C). CCR5 and CCR3 controls to identify gating boundaries for CCR3^+^ and CCR5^+^ T cells. (D). CXCR3 and CCR10 controls to set the gating for CXCR3^+^ and CCR10^+^ T cells. (E). Integrin beta 7 and CLA controls to set the gating to identify either gut homing or skin homing T cells.

## Epitope specific consideration

### CD3: this molecule is a lineage marker for T cells and highly expressed on T cells. Antibodies against this marker are available conjugated to all the fluorochromes. We decided to use an antibody conjugated to a dim fluorochrome like Alexa Fluor 700 and leave more brilliant fluorochromes to other less expressed markers.

### CD4: like CD3 and CD45RA this marker is highly expressed on T cells. In the panel, we preferred to use an antibody conjugated to a dim fluorochrome like BUV395. The main advantage was that BUV395 spill-over into other channels is minimal and we could reserve brighter fluorochromes to less expressed markers.

### CD25: this molecule has a low-density expression on T cells (less than 1000 molecules per cell). Even after increase following cell activation or on regulatory T cells that express the highest level (not more than 4000 molecules per cell). We tested three different clones M-A251, BC96 and 2A3 and found that M-A251 and 2A3 were giving a better staining. Because in this panel CD25 expression in combination with CD127 is crucial to separate regulatory from conventional T cells, we decided to use an antibody associated to a bright fluorochrome. We used the clone M-A251 conjugated to PE-CF594 (not available for clone 2A3) that in combination with the yellow laser excitation (instead of the blue laser) makes it one of the brightest fluorochromes available.

### CD127: this marker is not expressed at high density on T cells. However, like CD25, a sufficient separation between cells expressing high and low level of this molecule is necessary to clearly separate regulatory from conventional T cells. We decided to combine this marker with a bright fluorochrome like BV786 and tested different clones to obtain the best cell separation. Although results were very similar, clone HIL-7R-M21 gave the best stain index.

### CD45RA: the use of this marker is functional for the separation of both conventional T cells (e.g., naïve, central memory, effector memory, etc.) and regulatory T cells (naïve, memory subsets). This antigen has a high-density expression on cell surface (comparable to CD3, CD4 and CD8), to avoid issues due to SSE, we chose an antibody conjugated to a moderately bright fluorochrome like BUV496.

### CCR3 (CD193): this antigen is expressed on memory subsets of both T_conv_ and T_reg_ cells. It is used in this panel to identify cells trafficking to the upper airway mucosa. We combined this marker with BV510 because was one of the remaining fluorochrome available. Due to the low expression of this marker on T cells, we evaluated the performance of the antibody associated to BV510 on total lymphocytes. Results revealed the antibody working very well to separate positive and negative cells.

### CCR5 (CD195): this marker is expressed on activated memory subsets of T_conv_ and T_reg_ cells. It is involved in cell chemotaxis activation and trans endothelial migration during inflammation. It can be used in combination with CXCR3 to identify a small subset of cells accumulating in inflammatory lesions in different autoimmune disorders. To allow a good subset separation we chose to use an antibody conjugated to a very bright fluorochrome (especially when excited by a yellow laser) like PE-Cy7.

### CCR7 (CD197): the staining of this molecule can be used to identify different cell subsets. It is usually used to describe cells homing to lymph nodes such as naïve or resting memory cells. The molecule has a low density on T cells and the correct choice of the fluorochrome associated to the antibody is crucial to obtain a clear separation from negative and positive cells. In this panel, its staining is necessary to clearly discriminate between T cell differentiation steps (e.g., naïve, central memory, effector memory, etc.). We decided to combine anti-CCR7 with the bright fluorochrome BV421.

### CD28: antibodies against this marker are available conjugated to different fluorochromes. Because the molecule does not have a very high expression on all the cells, we chose a relatively bright fluorochrome like BV711.

### CD95: this molecule is usually expressed by activated T cells and T_reg_ cells. In this panel it is mainly used to identify the very small subpopulation of T_SCM_ cells. To improve the definition of this cells and allow a good separation for gating we decided to associate the antibody to a bright fluorochrome. We decided to use an antibody conjugated to the bright tandem-dye BUV737. This fluorochrome is excited by ultraviolet laser, but due to the excitation of the acceptor dye by the red laser, it can also produce spill-over into Alexa Fluor 700 channel. In this panel Alexa Fluor 700 is associated to anti-CD3 antibody and we did not observe any spill-over issues.

### CCR4 (CD194): this antigen can be used not only to define T_H_ subsets but also to separate antigen experienced T_reg_ cells from naïve. Its density on T cell surface is not very high and we decided to use in combination with a bright fluorochrome to allow a good separation of cell subsets when used in combination with CCR6. We tested the clone 1G1 with different fluorochromes (PE/Cy7 and BV605) with similar results. We chose to use the clone 1G1 conjugated to BV605 because when tested in combination with anti-CCR6 APC gave a good resolution.

### CCR10: this molecule can be found highly expressed in many disorders, especially in skin and intestinal diseases. In cells homing to the skin, it is often co-expressed with CCR4 and CLA. Because of its role in supporting the inflammatory response in this tissue, its blockade may attenuate skin inflammation. The anti-CCR10 clone 314305 (available only from R&D System) has been reported by other authors to provide a good resolution and successfully implemented conjugated to bright fluorochromes like PE and APC in OMIP-18 and OMIP-42 respectively. Unfortunately, it is currently available conjugated to a limited number of bright fluorochromes which in the iterative process used for the design of this panel, were already associated to other markers. Instead, we tested the clone 1B5 conjugated to BB515 and PerCP-Cy5.5 and obtained similar good results. We chose PerCP-Cy5.5 for this panel.

### CXCR3 (CD183): it is not a highly expressed marker, but it is crucial to identify T_H_ subsets. It can be also used in combination with CCR5 to evaluate changes of T cells infiltrating inflammatory lesions in autoimmune diseases. We decided to combine it with a bright fluorochrome, and tested antibodies conjugated to PE and PE-Cy5 with similar results. Due to limited availability of antibodies conjugates with PE-Cy5 and to leave PE fluorochrome available for other weak markers we chose to use CXCR3 with PE-Cy5 which in combination with the excitation by the yellow laser (instead of the blue laser) makes it one of the brightest fluorochrome available.

### CCR6 (CD196): this molecule has an important role to recruit T_reg_ cells and pro-inflammatory T_H_17 cells to the sites of inflammation. Its expression is required for the normal migration of these cells to the intestines where they play a part in regulating inflammatory processes. Staining of CCR6 requires a bright fluorochrome to allow a good separation from negative cells in a CCR4-CCR6 double stained sample. In this panel this combination of markers is crucial to the following T_H_ subset separation. We tested two different clones (G034E3 and QA17A37) with good results, but we decided to use the clone G034E3 associated with APC because allowed a better cell separation.

### CXCR5 (CD185): this molecule is associated to cells trafficking to follicles of spleen and Peyer patches. It is highly expressed on B cells and on T and T_reg_ cells controlling B cell activation. However, in contrast to B cell, this marker has a very weak expression on T cells. We decided to combine it with a bright fluorochrome to identify T_FH_ and T_FR_ cells. We tested the clone J252D4 with PE and APC. PE showed a higher resolution and was chosen for this panel.

### INTEGRIN BETA 7: this molecule is associated with the integrin alpha 4 chain which is expressed on cells trafficking to gut-associated lymphoid tissues. The complex of these two integrins interacts with the cell surface adhesion molecules MADCAM1 which is normally expressed by the vascular endothelium of the gastrointestinal tract. In this panel it is used to identify gut-homing T cells. We chose to use the antibody conjugated to BV650 because was one of the remaining bright fluorochrome available.

### CLA: Cutaneous lymphocyte-associated antigen (CLA) is co-expressed with CCR4 and CCR10 on lymphocytes with the capacity to traffic to inflamed skin. CLA mediates the rolling of T cells over activated endothelial cells expressing E-selectin. In our hands the anti-CLA clone HECA-452 conjugated to FITC worked always well and we included it in this panel.

### VIABILITY DYE: Fixable Viability Stain 780 is excited with red laser and has a fluorescence emission maximum of 780 nm. Because antibodies conjugated to tandem-dyes emitting in the same channel (e.g., APC-Cy7) have been described to be unstable and with spill-over into APC, we decided to use this channel as a ‘dump-channel’ and this dye for the exclusion of all dead cells.

# **Cross-references to relate panels**

# There is no evidence of highly related panels already published elsewhere. Between OMIPs, this panel shares the objective to use only surface markers to describe multiple human T cells subsets in peripheral blood with OMIP-017, -018, -30 and -42. OMIP-18 is the only panel that uses a large number of chemokine receptors like this panel to identify all T helper cell subsets. However, like OMIP-17, OMIP-18 does not include markers to characterise regulatory T cells (i.e., CD25 and CD127). Besides their capacity to analyse multiple T cell populations, OMIP-30 includes activation and proliferation markers, and OMIP-42 has the capacity to enumerate non-T cell populations like monocytes, DC, NK and B cells. Unlike this panel, none of the OMIPs described above can investigate cell trafficking to non-lymphoid tissues (i.e., skin, gut, or airway mucosa).

1. **Exact staining protocol**

**Materials**

- Ficoll-Paque™ Plus solution (cat. N. 17-1314-01, Cytiva)
- Dulbecco Phosphate Buffered Saline (DPBS) – (cat. N. 14190144, Gibco)
- Heat-inactivated Human Serum (cat. N. H4522, Sigma-Aldrich)
- Staining buffer: DPBS, 0.5 % (w/v) BSA (cat. N. A7906-500G, Sigma-Aldrich), 2mM EDTA (cat. N. 15575020, Gibco)
- BD Brilliant Horizon™ Stain buffer (cat. N. 563794, BD Biosciences)
- Falcon 5 ml round-bottom polystyrene tubes (12 x 75 mm) - (cat. N. 352052, Falcon)

**Note** Peripheral blood sample should not be older than 8 hours.

Whole blood should be supplemented with anticoagulants (e.g., EDTA, citrate, heparin)

**Protocol**

1. Prepare a 15 ml conical tube containing 4 ml of Ficoll-Paque™
2. Dilute whole blood 2-4 times with DPBS and carefully layer the diluted blood over Ficoll-Paque™.
3. Centrifuge for 20 minutes at 600 x *g* in a swinging bucket rotor without acceleration and brake at 20°C
4. Carefully transfer the complete PBMC layer using a sterile Pasteur pipette to a new 15 ml conical tube.
5. Wash cells in 10 ml DPBS and centrifuge at 600 x *g* for 10 minutes at 20°C
6. Discard supernatant and resuspend cells in residual DPBS
7. Wash cells in 10 ml DPBS and centrifuge at 200 x *g* for 10 minutes at 20°C to remove platelets
8. Discard supernatant and resuspend cells in 5 ml of DPBS
9. Count cells on haemocytometer and resuspend cells in DPBS at 1 x 10^6^/ml
10. Transfer 1 ml of cell suspension in a new round-bottom tube (*keep the rest of the cells for unstained and compensation controls*)
11. Centrifuge at 300 x *g* for 10 minutes at 20°C
12. Prepare a solution of BD Horizon Fixable Viability Stain 780 in DPBS (1:1000 dilution)
13. Discard supernatant and resuspend cells in 1 ml of Fixable Viability Stain 780 solution
14. Incubate cells at 37°C for 7 minutes in the dark
15. Wash cells in 3 ml of staining buffer solution containing 10% human serum
16. Centrifuge at 300 x *g* for 10 minutes at 20°C
17. Discard supernatant
18. Wash cells in 3 ml of staining buffer solution containing 10 % human serum
19. Centrifuge at 300 x *g* for 10 minutes at 20°C
20. Discard supernatant
21. Prepare Surface antibody mix solution containing:
    1. Staining buffer solution with 10 % human serum
    2. Brilliant Stain buffer solution (50 μl/sample)
    3. Antibodies for surface staining
22. Resuspend cells in 100 μl of Surface antibody mix solution
23. Incubate cells at 37°C for 20 minutes in the dark
24. Wash cells adding 1 ml of staining buffer solution
25. Centrifuge at 300 x *g* for 10 minutes at 20°C
26. Discard supernatant
27. Wash cells adding 1 ml of staining buffer solution
28. Centrifuge at 300 x *g* for 10 minutes at 20°C
29. Discard supernatant
30. Resuspend cells in 400 μl of staining buffer solution
31. Acquire samples on Flow cytometer analyser

# **Instrument configuration – Online Table 1**

| **Laser** | **Laser** | **Laser** | **Spectral range** | **Optical filters** | | **Fluorochrome/** |
| --- | --- | --- | --- | --- | --- | --- |
| **wavelength** | **power** | **type** | **for detector** | **Dichroic** | **Band pass** | **Reagents** |
| **[nm]** | **[mW]** |  | **[nm]** | **filter** |  | **used** |
| **355nm** | 65mW | DPSS | 722-757 | 690LP | 740/35 | **BUV737** |
|  |  |  | 505-525 | 410LP | 515/20 | **BUV496** |
|  |  |  | 365-393 | blank | 379/28 | **BUV395** |
| **405nm** | 100mW | DPSS | 750-810 | 750LP | 780/60 | **BV786** |
|  |  |  | 685-735 | 685LP | 710/50 | **BV711** |
|  |  |  | 650-670 | 630LP | 660/20 | **BV650** |
|  |  |  | 600-620 | 570LP | 610/20 | **BV605** |
|  |  |  | 500-550 | 505LP | 525/50 | **BV510** |
|  |  |  | 425-475 | blank | 450/50 | **BV421** |
| **488nm** | 100mW | DPSS | 685-735 | 640LP | 710/50 | **PerCP-Cy5.5** |
|  |  |  | 515-545 | 505LP | 530/30 | **AF488** |
|  |  |  | 483-493 | blank | 488/10 | **SSC** |
| **561nm** | 50mW | DPSS | 750-810 | 750LP | 780/60 | **PE-Cy7** |
|  |  |  | 650-670 | 630LP | 660/20 | **PE-Cy5.5** |
|  |  |  | 600-620 | 600LP | 610/20 | **PE-CF594** |
|  |  |  | 574-589 | blank | 586/15 | **PE** |
| **640nm** | 40mW | DPSS | 750-810 | 750LP | 780/60 | **Live/Dead NIR** |
|  |  |  | 707-753 | 690LP | 730/45 | **AF700** |
|  |  |  | 663-677 | blank | 670/14 | **APC** |

This panel was optimized for the LSR-Fortessa (BD Biosciences). The 620/10 band pass for PE-CF594 was chosen over the standard 610/20 to minimize spill-over from QD605. AF = Alexa Fluor, APC = Allophycocyanin, BV = Brilliant Violet, Cy = cyanine, eF = eFluor, DPSS = Diode Pumped Solid State, Live/Dead NIR (Near Infra-Red) = Fixable Viability Stain 780, PE = R-phycoerythrin, PerCP = Peridinin chlorophyll, QD = Quantum Dot

# **Reagent Information – Online Table 2**

| Marker | Fluorochrome | Clone | Vendor | Catalog number | Volume used  (µL/100 µL) |
| --- | --- | --- | --- | --- | --- |
| CD3 | AF 700 | UCHT1 | BD Biosciences | 557943 | 1 |
| CD4 | BUV 395 | SK3 | BD Biosciences | 563550 | 1 |
| CD25 | PE/CF594 | M-A251 | BD Biosciences | 562403 | 3 |
| CD127 | BV 786 | HIL-7R-M21 | BD Biosciences | 563324 | 3 |
| CD45RA | BUV 496 | HI100 | BD Biosciences | 750258 | 1 |
| CD28 | BV 711 | CD28.2 | Biolegend | 302948 | 5 |
| CD95 | BUV 737 | DX2 | BD Biosciences | 612790 | 1 |
| CCR3 | BV 510 | 5E8 | Biolegend | 310722 | 0.5 |
| CCR4 (CD194) | BV 605 | 1G1 | BD Biosciences | 562906 | 5 |
| CCR5 | PE/Cy7 | J418F1 | Biolegend | 359108 | 0.5 |
| CCR6 (CD196) | APC | G034E3 | Biolegend | 353416 | 3 |
| CCR7 (CD197) | BV 421 | G043H7 | Biolegend | 353208 | 7 |
| CCR10 | PerCP/Cy5.5 | 1B5 | BD Biosciences | 564772 | 1 |
| CXCR3 (CD183) | PE/Cy5 | 1C6/CXCR3 | BD Biosciences | 551128 | 5 |
| CXCR5 (CD185) | PE | J252D4 | Biolegend | 356904 | 1 |
| Integrin Beta 7 | BV 650 | FIB504 | BD Biosciences | 564285 | 0.25 |
| CLA | FITC | HECA-452 | Biolegend | 321306 | 5 |
| Fixable Viability Stain 780 | Live/Dead Near Infra-Red | n/a | BD Biosciences | 565388 | 1 |
| AF = Alexa Fluor, APC = Allophycocyanin, BV = Brilliant Violet, BUV = Brilliant Ultraviolet, Cy = cyanine,  FITC = Fluorescein isothiocyanate, PE = R-phycoerythrin, PerCP = Peridinin-Chlorophyll-Protein. | | | | | |

# **Antibody Titration - Online Figure 4**


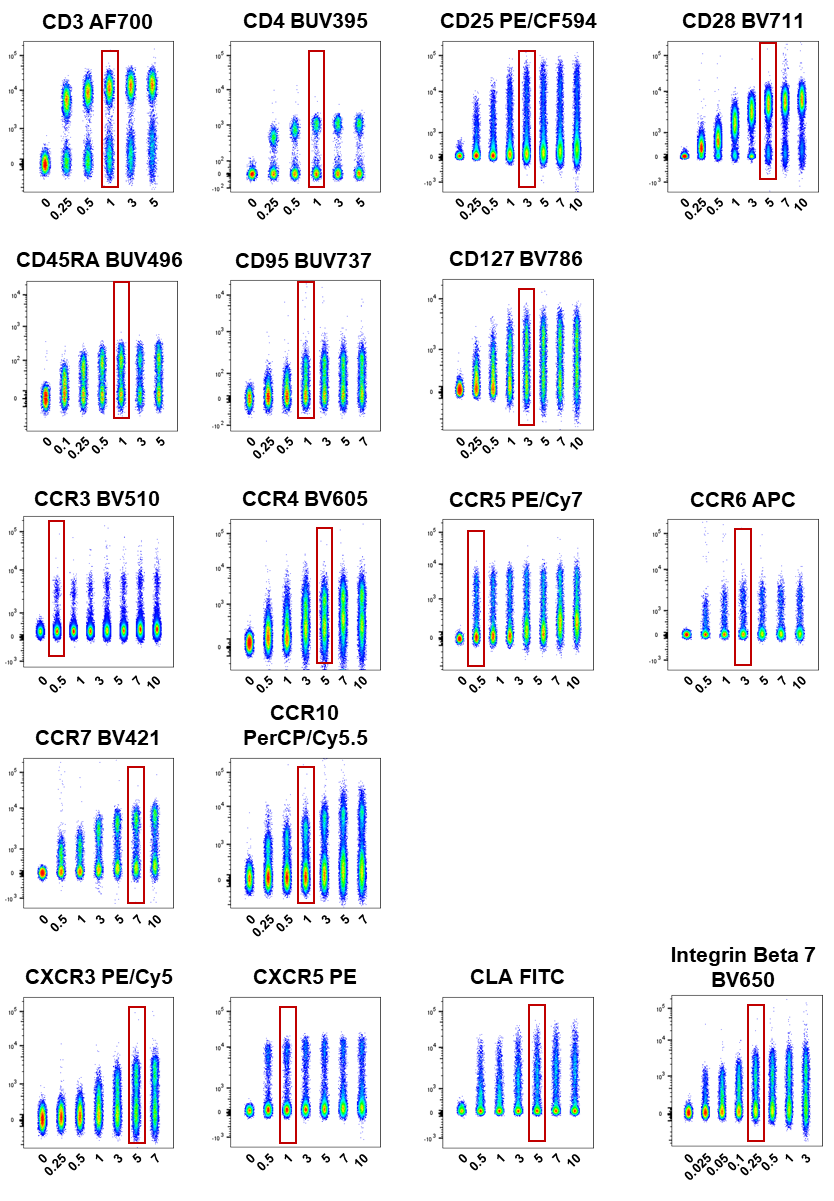


**Online** **Figure 4. Antibody titration.** All the antibodies used in the panel were titrated on the same number of human PBMCs isolated from a healthy donor (1x10^6^ cell/test). Individual ‘.fcs’ files were concatenated in a single pseudo-colour dot-plot with the aim of visualising all the titrations at the same time. Antibody concentrations (expressed in µL/100µL) were arranged from the lowest (unstained) to the highest along the x-axis starting on the left of each graph. Data are representative from at least two independent experiments. The chosen concentration of each antibody was highlighted with a red rectangle and reported in Table 2.

# **Online Table 3a. T_conv_ cell populations identified by the panel**

| **Population** | **Gated on** | **Type** | **Subset** | **Phenotype** |
| --- | --- | --- | --- | --- |
| **CD4^+^ T_conv_** | Live^+^/CD3^+^/CD4^+^ CD127^+/low^/CD25^low/-^CD45RA^+^ | Naïve | n/a | CCR7^+^ CD28^+^ CD95^-^ |
|  |  | Memory | T_SCM_ | CCR7^+^ CD28^hi^ CD95^+^ |
|  |  |  | T_E or_T_EMRA_ | CCR7^-^ CD28^-^ |
|  |  |  |  |  |
|  |  | Memory | T_CM_ | CCR7^+^ CD28^+^ |
|  | Live^+^/CD3^+^/CD4^+^  CD127^+/low^/CD25^low/-^CD45RA^-^ |  | T_TM_ | CCR7^-^ CD28^+^ |
|  |  |  | T_EM_ | CCR7^-^ CD28^-^ |
|  |  |  |  |  |
|  |  | Helper | T_FH_ | CXCR5^+^ |
|  |  |  |  |  |
|  |  |  | T_H_1 | CCR4^-^ CCR6^-^ CCR10^-^ CXCR3^+^ |
|  |  |  |  |  |
|  |  |  | T_H_2 | CCR4^+^ CCR6^-^ CCR10^-^ CXCR3^-^ |
|  |  |  |  |  |
|  |  |  | T_H_9 | CCR4^-^ CCR6^+^ CCR10^-^ CXCR3^+^ |
|  |  |  |  |  |
|  |  |  | T_H_17 | CCR4^+^ CCR6^+^ CCR10^-^ CXCR3^‑^ |
|  |  |  |  |  |
|  |  |  | T_H_22 | CCR4^+^ CCR6^+^ CCR10^+^ CXCR3^-^ |
|  |  |  |  |  |
|  |  |  | GM-CSF^+^ T_H_ | CCR4^+^ CCR6^-^  CCR10^+^ CXCR3^-^ |
|  |  |  |  |  |
| Note: T_conv_ = conventional T cell; T_SCM_ = Stem cell memory T cell; T_CM_ = Central memory; T_TM_ = Transitional memory; T_EM_ = Effector memory; T_E_ or T_EMRA_ = Terminal effector; T_FH_ = T follicular helper cell; T_H_ = T helper cell. | | | | |

# **Online Table 3b. Regulatory T cell populations identified by the panel**

| **Population** | **Gated on** | **Subset** | **Phenotype** |
| --- | --- | --- | --- |
| **CD4^+^ T_reg_ cell** | Live^+^/CD3^+^/CD4^+^/  CD25^hi^CD127^low^ | Naïve (Pop I) | CD25^+^ CD127^low^ CD45RA^+^ |
|  |  |  |  |
|  |  | Effector (Pop II) | CD25^hi^ CD127^low^ CD45RA^-^ |
|  |  |  |  |
|  |  | Memory/Cytokine-producing (Pop III) | CD25^+^ CD127^low^ CD45RA^-^ |
|  |  |  |  |
| **CD4^+^ T_reg_ helper-like** | Live^+^/CD3^+^/CD4^+^/  CD25^hi^CD127^low^ CD45RA^-^ | T_FR_ | CXCR5^+^ |
|  |  |  |  |
|  |  | T_H_1-like | CCR4^-^ CCR6^-^ CCR10^-^ CXCR3^+^ |
|  |  |  |  |
|  |  | T_H_2-like | CCR4^+^ CCR6^-^ CCR10^-^ CXCR3^-^ |
|  |  |  |  |
|  |  | T_H_9-like | CCR4^-^ CCR6^+^ CCR10^-^ CXCR3^+^ |
|  |  |  |  |
|  |  | T_H_17-like | CCR4^+^ CCR6^+^ CCR10^-^ CXCR3^‑^ |
|  |  |  |  |
|  |  | T_H_22-like | CCR4^+^ CCR6^+^ CCR10^+^ CXCR3^-^ |
|  |  |  |  |
| Note: T_reg_ = regulatory T cell; T_FR_ = T follicular regulatory cell; T_H_ = T helper cell. | | | |

# **Brief description of sample data files that have been uploaded electronically.**

Exemplary data files (.fcs format) from 3 individuals were submitted to [http://flowrepository.org](http://flowrepository.org/)

http://flowrepository.org/id/FR-FCM-Z4C6.

URL: <https://flowrepository.org/id/RvFrht4UEL8oNrHFtYFy8Ru0WJRhnnoHuSIMrcGsjmMW9hXn2SCJHYjekghh1TT1>

Samples were acquired on a BD LSR II Fortessa equipped with 5 lasers (see Online Table 1 for details). BD™ CS&T beads were used to setup the flow cytometer baseline and the same day of the sample acquisition to provide a standardised method to perform quality control of the instrument’s optics, electronics, and fluidics, and for adjusting fluorescence compensation. The three stained samples in the flow repository show the cell distribution of two healthy donors and one individual with allergic rhinitis.
